# The practice of oral feeding after total laryngectomy by Brazilian head and neck surgeons^☆^^☆☆^

**DOI:** 10.1016/j.bjorl.2025.101644

**Published:** 2025-06-04

**Authors:** Johanna Benali, Tareck Ayad, Fabio Pupo Ceccon, Carlos Chiesa-Estomba, Jerome R. Lechien, Mateus Morais Aires, Leonardo Haddad, Gerrit Viljoen, Nicolas Fakhry

**Affiliations:** a Young-Otolaryngologists of the International Federation of Otorhinolaryngological Societies (YO-IFOS), Paris, France; b Aix-Marseille Université, La Conception University Hospital, Department of Oto-Rhino-Laryngology Head and Neck Surgery, Marseille, France; c Centre Hospitalier de l’Université de Montréal, Division of Otolaryngology Head and Neck Surgery, Montreal, Québec, Canada; d Universidade Federal de São Paulo, Escola Paulista de Medicina, Departamento de Otorrinolaringologia - Cirurgia de Cabeça e Pescoço, São Paulo, SP, Brazil; e Hospital Universitario Donostia, Department of Otorhinolaryngology - Head and Neck Surgery, San Sebastian, Spain; f University of Mons (UMONS), Research Institute for Health Sciences and Technology, Department of Anatomy and Experimental Oncology, Mons, Belgium; g Elsan Hospital, Paris, France; h Foch Hospital Paris Saclay University, Department of Otorhinolaryngology and Head and Neck Surgery, Paris, France; i Aix-Marseille Université, Sciences Economiques & Sociales de la Santé & Traitement de l’Information Médicale, Marseille, France

**Keywords:** Laryngectomy, Postoperative care, Postoperative complications

## Abstract

**Objective:**

To evaluate the initiation of oral feeding in patients following TL by a group of Brazilian head and neck surgeons.

**Methods:**

Online survey – 75 responders from Brazil.

**Results:**

40.5% of the respondents introduced water and 41.9% of them introduced liquid diets between days 7 and 9 after TL without surgical complications or previous radiotherapy. Semi-solid feeds were started between days 10 and 14 in 47.3% of the patients and a free diet was begun after day 15 in 79.7% of them. There was statistically significant difference in the initiation of liquid feeds between different groups of TL patients, with earlier initiation in TL patients who had not undergone prior radio(chemo)therapy (*p* = 0.01419), with even greater differences when complex closure of the pharynx was needed (*p* = 0.00001), but not regarding the moment of a free diet introduction.

**Conclusion:**

Most respondents in this Brazilian cohort prefer to wait at least 7-days before beginning oral feeding after TL without previous radiotherapy or surgical complications, with a significant number of respondents postponing feeds in patients who had undergone salvage TL and pharyngolaryngectomy.

**Level of evidence:**

Level IV.

## Highlights

• Oral feeding is usually offered after 7 days of surgery for uncomplicated cases without previous radiation therapy or extension of surgery to the hypopharynx.
• The enteral feeding is kept up to 15 days for complicated cases, salvage surgery of pharyngolaryngectomy cases.
• Patients offered oral feeding before the 10th day of surgery are discharged from the hospital within 14 days of surgery.

## Introduction

Curative management of malignant tumors of the larynx and hypopharynx vary according to disease stage and location. Total Laryngectomy (TL) is a radical and effective procedure in the therapeutic management of laryngeal and hypopharyngeal advanced tumors compared with organ preservation protocols with chemotherapy and radiotherapy in selected patients. TL and the Pharyngolaryngectomy (PL) are indicated as follows: advanced Tumors (T4a), significant laryngeal dysfunction after radio(chemo)therapy, contraindications to curative radio(chemo)therapy, and recurrence after partial laryngectomy or radio(chemo)therapy treatment.^1^ However, patients undergoing TL are at risk of multiple postoperative complications,^2^ of which Pharyngocutaneous Fistula (PCF) is the most frequent, with incidence varying from 10% to 34%.^3^ PCF usually occurs between days-5 and -6 after surgery, prior to the reintroduction of feeds. Because of the presence of edema and skin hyperemia, PCF is not always easy to diagnose in the first days of onset. This complication is related to deep neck infections and leads to increased morbidity and mortality due to revision surgeries, prolonged length of hospital stay, delays in starting adjuvant therapy and the danger associated with carotid blowout.^3^ Despite the variability between studies, important risk factors include a poor surgical technique, preoperative malnutrition, postoperative hematoma, compromised surgical margins, previous radiotherapy, advanced tumor stage, hypopharyngeal tumors, complex reconstruction of the remaining pharynx using regional or free flaps, and postoperative hemoglobin level < 99 g/L.^4^, ^5^, ^6^, ^7^, ^8^, ^9^, ^10^

During the first half of the last century, the restart of oral feeding after TL was considered one of the major risk factors for PCF and it was thus initiated only 10-days or more after surgery.^11^, ^12^ Despite these assumptions, multiple studies have addressed early feeding to determine whether it could be a safe practice considering the daily volume of saliva humans produce and swallow.^13^, ^14^, ^15^, ^16^, ^17^, ^18^, ^19^, ^20^, ^21^, ^22^, ^23^, ^24^ Sousa et al.^23^ conducted a study with 89 patients and compared two groups randomly allocated: Group 1 (44 patients) – early oral feeding beginning 24 h after surgery and Group 2 (45 patients) – late oral feeding staring at day-7 after surgery. They observed 10 and six salivary fistulas in Groups 1 and 2, respectively. There was no statistically significant difference in the incidence of PCF between the two groups. The only variables associated with PCF were the involvement of surgical margins and invasive carcinomas. Additionally, early feeds could have several benefits, including improved quality of life, reduced requisite for postoperative care, and shortened length of hospital stay. All these benefits can ultimately lead to reduced costs of management and care.^18^

This study aimed to investigate the practices of restarting feeds applied in centers around the world, study the differences in approaches between the regions, and evaluate the factors influencing the delay in oral feeding after TL. The worldwide results were recently published.^19^ Since many answers from Brazilian head and neck surgeons were collected from this survey, a separate analysis was performed and is herein presented to evaluate the Brazilian data, followed by a comparative evaluation with the data obtained from globally when applicable.

## Methods

This research was developed using an iterative method. The study group includes head and neck surgeons from all continents. The questions were chosen to investigate the management of TL patients, especially regarding oral intake in different circumstances. The questionnaire was prepared in the SurveyMonkey platform (San Mateo, California, USA). The first version of the questionnaire was prepared by a committee comprising certified otolaryngologists and/or head and neck surgeons from five continents and 14 countries (Argentina, Belgium, Brazil, Canada, Columbia, Italy, France, Lebanon, New Zealand, Singapore, Spain, South Africa, Thailand, and the USA). The survey was revised and completed based on their comments. The final version of the survey included 29 questions divided into seven sections: general information, patient nutritional status assessment, technical considerations (type of mucosal sutures and methods followed to ensure the absence of PCF before starting feeds), and the moment of the beginning of oral feeding was studied in four scenarios, designated as Cases 1 to 4:

–Patients who underwent TL without pharyngectomy, without prior radiotherapy and with primary mucosal closure (Case 1);
–Patients who underwent Salvage Laryngectomy (SL) after previous radiotherapy, without pharyngectomy and with primary mucosal closure (Case 2);
–Patients who underwent TL with concomitant pharyngectomy (total laryngopharyngectomy) for a tumor involving the hypopharynx, without previous radiotherapy, with the concurrent use of a pedicled or free flap to reconstruct a pharyngeal mucosal defect (Case 3);
–Patients who underwent SL associated with pharyngectomy (total laryngopharyngectomy) for a tumor involving the hypopharynx after prior radiotherapy with the use a pedicled or free flap to reconstruct a pharyngeal mucosal defect (Case 4).

Ethical Committee approval: the Institutional Review Board approved the protocol (nº 2021-04-08-03).

Survey spread: a link to the survey was e-mailed four times (this included an initial e-mail followed by three reminders) to laryngologists and head and neck surgeons worldwide from February to April 2021, as well as to members from Brazilian Society of Head and Neck Surgery during the same period. Each participant was allowed to complete the survey only once.

Response Collection and Statistical Analysis: responses were collected anonymously, and incomplete responses were excluded from the analysis. The city and institution of every respondent were identified. Only responses from Brazil were used in this study. As head and neck surgeons may work in different institutions (public and private practice) in Brazil, each answer was considered individually, and not as an institutional response.

### Statistical analysis

Categorical variables were described as number and percentage. They were compared by the Chi-Squared test, the Fisher test, or the Chi-Squared test with p-value simulation depending on the application conditions. By default, this simulation was performed on 2000 random selections. The tests were performed in a two-sided situation and were considered statistically significant when *p* ≤ 0.05. Statistical analyses were conducted using the RStudio Desktop 1.4.1106 software.

## Results

As the diffusion method was used, it was not possible to identify how many surgeons received the invitation to answer the questionnaire. However, we estimate that the questionnaire reached 850 head and neck specialists from Brazil. A total of 74 complete questionnaires were received, and one incomplete questionnaire was excluded from the study. Of the 74 surgeons who answered the questionnaire, 37 practiced in hospitals linked to universities, 37 in institutions dedicated to cancer, 18 in regional hospitals, and 46 in private institutions, which confirmed that many Brazilian surgeons work in more than one type of hospital setting, justifying the use of considering each respondent individually in this study. Twenty-seven (36.5%) surgeons informed that they perform less than 10 TL per year, 22 (29.7%) between 10 and 19 procedures per year, and 25 (33.8%) more than 20 procedures a year. Thirty-nine (52.7%) participants reported performing less than 25% SL after radio(chemo)therapy, 22 (29.7%) between 25% and 50%, and 13 (17.5%) more than 50%. This shows that the respondents represent a diverse sample of the daily practices of surgeons working in several types of hospitals, together with a differing volume of procedures, and a varying proportion of salvage surgery performed among these operations.

### Organization of patient management

Regarding the preoperative evaluation of patient nutritional status, the body mass index was used by 36 (48.6%) of the surgeons, albumin and pre-albumin measurement by 33 (44.6%), percentage of weight loss by 33 (44.6%), and nutritional assessment questionnaires by 32 (43.2%) of them. Twelve (16.2%) specialists reported not assessing patient nutritional status preoperatively. Forty-seven (63.5%) surgeons had dieticians in their multidisciplinary team to manage the nutrition of patients, 22 (29.7%) counted on dieticians only in cases of confirmed malnutrition, and 5 (6.7%) have never had the collaboration of dieticians. During the postoperative period, most of the participants, 73 (98.6%), reported the use of a feeding tube for nutritional support, 57 (77%) informed that they only use a nasogastric feeding tube, 2 (2.7%) reported the use of gastrostomy only, 14 (18.9%) used nasogastric feeding tube and gastrostomy, and 1 (1.3%) used intravenous infusion only. These results indicate that nasogastric feeding tubes were the most frequently used devices for nutritional support following surgery.

### Oral feeding after surgery

**Fig. 1:**
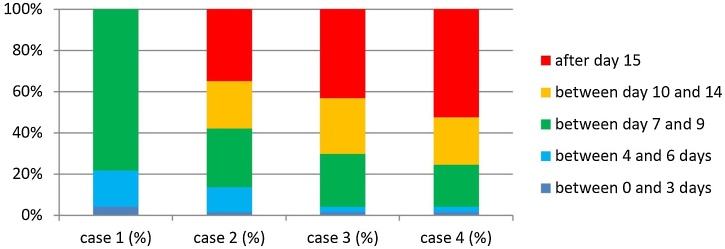
Timing of introduction of hydration with water and day of oral feed × percentage of patients, with significant difference between Case 1 × Others, *p* < 0.05.

For Case 1 patients (TL without pharyngectomy, without prior radiotherapy and with primary mucosal closure), 78.4% of the surgeons initiated oral hydration with water after day-7. A liquid diet (e.g., juice, milk) was started after day-7 by 41.9% and after day-10 by 45.9%. Semi-solid food (e.g., mixed food, puree) was started by 47.3% between day-10 and -14 and by 39.2% after day-15. A free diet was initiated by 79.7% after day-15. (Fig. 1).

**Fig. 2:**
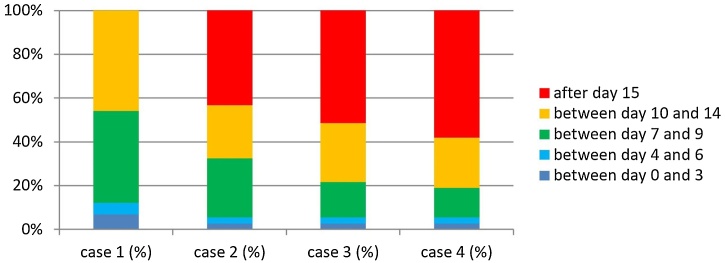
Timing of introduction of liquid diet and day of oral feed × percentage of patients, with significant difference between Case 1 × Others, *p* < 0.05.

In Case 2 patients (SL after prior radiotherapy, without pharyngectomy, with primary mucosal closure and without complications), 28.4% of the respondents began oral hydration with water after day-7, and most of them after day-10 (58.1%). A liquid diet (e.g., juice, milk) was started by 57.5% after day-10. Semi-solid food was allowed by 31.1% between day-10 and 14, and most of them (64.9%) after day-15. A free diet was introduced by 82.4% after day-15 (Fig. 2).

**Fig. 3:**
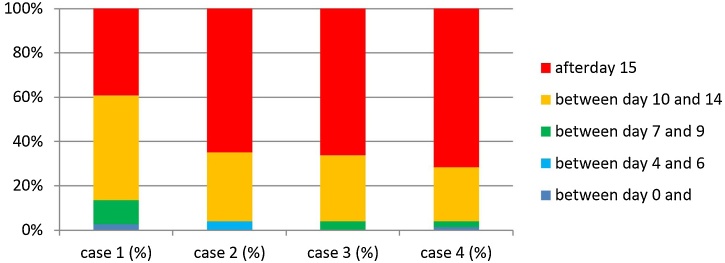
Timing of introduction of semi-solid diet and day of oral feed × percentage of patients, with significant difference between Case 1 × Others, *p* < 0.05.

In Case 3 patients (TL associated with pharyngectomy [Total Laryngopharyngectomy]) for a tumor involving the hypopharynx, without prior radiotherapy, with the concurrent use of a pedicled or free flap to reconstruct a pharyngeal mucosal defect), the application of a more conservative practice was observed, with 70.2% of the respondents reintroducing water after day-10 and liquids (e.g., juice, milk) being allowed by 78.4% after day-10. A semi-solid diet was introduced by 66.2% after day-15 and a free diet by 91.9% after day-15 (Fig. 3).

**Fig. 4:**
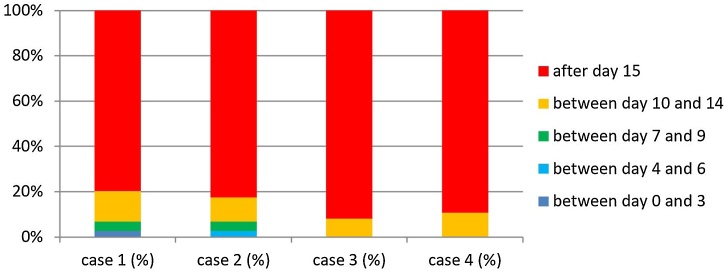
Timing of introduction of free diet and day of oral feed × percentage of patients, without significant difference between Case 1 × Others.

For Case 4 patients (SL associated with pharyngectomy for a tumor involving the hypopharynx after prior radiotherapy with the use a pedicled or free flap to reconstruct a pharyngeal mucosal defect), an even more conservative approach was observed. Oral feeds with water were started by 52.7% of the surgeons after day-15 and liquids (e.g., juice, milk) by 81% after day-10. Semi-solid food was introduced by 71.6% after day-15 and a free diet by 89.2% after day-15 (Fig. 4).

We compared the times of beginning feeds in Case 1 with those in the other groups (Cases 2, 3 and 4) to evaluate the conduct related to feed when surgeons were faced with more severe disease that required more extensive surgeries. The Chi-Squared test was applied, and the following results were obtained. Comparing the days following surgery when oral water was reintroduced, there were significant differences between Case 1 and Case 2, between Case 1 and Case 3, and between Case 1 and Case 4. The comparative study regarding the timing to reintroduce oral liquid diet feeds revealed significant differences between Case 1 and Case 2, Case 1 and Case 3, and Case 1 and Case 4. The comparative statistical analysis for the reintroduction of a semi-solid diet showed significant differences between Case 1 and Case 2, Case 1 and Case 3, and Case 1 and 4. The initiation of a free diet was not significant in the comparisons between Case 1 and the other cases.

About the surgical technique of mucosal closure of the pharynx in Case 1 and the rate of PCF. Most surgeons made use of more than one type of closure: stapler closure (35); T-shape interrupted sutures (30); T-shape running sutures (21); vertical (longitudinal) interrupted sutures (3); vertical (longitudinal) running sutures (8); horizontal (transverse) interrupted sutures (5); horizontal running sutures (6). The projected rate of PCF in TL was <10% for 17 (22.9%) of the respondents, 10%‒25% for 41 (55.4%), 25%‒50% for 14 (18.9%), and > 50% for 2 (2.7%) of them.

Surgeons used of the following methods to exclude PCF before oral feeds: 63.5% performed a diagnostic methylene blue test, 5.4% conducted a barium swallow test, and 63.5% used clinical observation alone (we considered that 63.5% of the participants did not perform any test, but if indicated, they would prefer using of the blue dye test more often than the barium test). Blue methylene tests were performed between days-4 and -6 by 18.9%, days-7 and -9 by 41.9%, and days-10 and -14 by 16.2% of the respondents.

The reported length of hospital stay after TL without complications was < 7-days for 62.2% (46/74), 8‒14-days for 33.8% (25/74), and 15‒21-days for 4% (3/74).

Participants were asked about the specific factors that would routinely lead them to postpone oral intake after TL, even with no postoperative complications. Among the proposed circumstances, previous radiotherapy was indicated by 41 participants (55.4%), previous radiotherapy with concurrent chemotherapy by 40 (54%), and the use of a pedicled or free flap to reconstruct a pharyngeal mucosal defect by 34 (45.9%). General illnesses, such as diabetes mellitus, were selected by 13 (17.6%) of the respondents, advanced age by 5 (6.7%), primary voice prosthesis during surgery by 3 (4%), and previous tracheostomy by 4 (4.7%). Eleven participants (4.8%) did not respond to this question.

Analysis of the time to start oral feeding showed no statistically significant difference between groups performing fewer than 10 TL per year and those with more than 10 per-year (Table 1). There was also no difference for salvage surgeries between groups of surgeons performing more than 25% of salvage surgery and those performing less than 25% (Table 2).

**Table 1:** Time to start oral feeding according to the number of procedures.

Time to begin oral feeding with water	< 10 TL/year	> 10 TL/year
Until day 6	28.6% (8)	17.4% (8)
Between day 7 and 9	28.6% (8)	50% (23)
Between day 10 and 14	25% (7)	19.6% (9)
After day 15	17.8% (5)	13.0% (6)

Time to start oral feeding according to the number of procedures; no statistically significant difference (Chi-Squared test, *p* = 0.331).

**Table 2:** Time to start oral feeding after salvage TL according to the proportion of salvage TL performed every year.

Time to start oral feeding with water after salvage laryngectomy	Surgeon performs < 25% Salvage TL	Surgeon performs > 25% Salvage TL
Until day 6	12.9% (5)	14.3% (5)
Between day 7 and 9	28.2% (11)	31.4% (11)
Between day 10 and 14	25.6% (10)	20.0% (7)
After day 15	33.3% (13)	34.3% (12)

Time to start oral feeding after salvage TL according to the proportion of salvage TL performed every year; no statistically significant difference (Chi-Squared test, *p* = 0.949).

Time to start oral feeds showed a difference that was not statistically significant between those reporting less than 10% rates of PCF and those reporting more than 10% (Table 3). Regardless of the type of oral intake, it was observed that participants who discharged patients within the first 14-days after surgery were more likely to allow oral intake before day-10 compared with those who kept patients in hospital for more than 14-days (Table 4).

**Table 3:** Rate of pharyngocutaneus fistulas and number of salvage total laryngectomies.

Rate of PCF	Surgeons < 25% Salvage TL	Surgeons > 25% Salvage TL
Until 10%	9	8
10−25%	25	16
> 26%	6	10

Rate of pharyngocutaneus fistulas; no statistically significant difference between surgeons with different number of salvage total laryngectomies (Chi-Squared test, *p* = 0.278).

**Table 4:** Time to start oral feeding with water according to length of hospital stay.

Time to start oral feed (water)	Until 7^th^ day	> 7^th^ day
until 6^th^ day	15.2% (7)	32.1% (9)
Between 7^th^ and 9^th^	41.3% (19)	39.3% (11)
Between 10^th^ and 14^th^	19.6% (9)	25.0% (7)
Ater 15^th^ day	23.9% (11)	3.6% (1)

Time to start oral feeding with water according to length of hospital stay; no statistically significant difference (Chi-Squared test, *p* = 0.072).

To summarize, most patient who underwent TL without pharyngectomy and without previous radiotherapy started oral hydration and liquids between days-7 and -10, semi-solid food was most often initiated between days-10 and -14, and a free diet was introduced after 15-days. There were further delays in feeding initiation in cases of previous radiotherapy or flap reconstruction.

There was no difference in the time of feeding initiation according to the number of procedures, the proportion of salvage surgery, or the declared rate of PCF. It was observed that a higher proportion of surgeons who discharged their patients within 14 postoperative days allowed oral intake before day-10 compared with those who keep their patients hospitalized for more than 14-days.

## Discussion

Online web research has recently emerged as an important tool to explore behaviors, shopping preferences, social aspects of life, and regional differences in various daily conducts and practices. Especially in the field of medicine, this research method can reach many individuals. This study is, subsequently, the result of an international online survey that aimed to explore the timing of the reintroduction of feeds after total laryngectomies. It was carried out from the data collected by this global survey that gathered 278 responses from surgeons from 59 different countries. These surgeons practiced in different types of institutions and performed a varying number of TL each year with a differing percentage of salvage operations.^19^ The number of respondents from Brazil was 75, and one was excluded because of incomplete answers. This large number of respondents was an important motivation to perform a separate analysis of Brazilian surgeons regarding the introduction of feeds after TL.

This study presents the conduct of a group of Brazilian surgeons in different scenarios involving TL from preoperative nutritional evaluation to hospital discharge. The postoperative management of patients who underwent TL is controversial when considering the initiation of feeding. The practice of early oral feeding following TL is not universally acknowledged despite evidence supporting its safety and benefits.

Many publications indicated that early oral feeding in the first 7-days after surgery is a safe practice that does not lead to an additional risk of PCF and may improve patient quality of life. It also avoids the initial or prolonged use of a nasogastric tube and reduces length of hospital stay and the medical costs of hospitalization.^17^, ^18^, ^19^, ^20^, ^21^, ^22^ On the other hand, a work published by Sousa^23^ points to concerns regarding the ability to provide adequate nutrition during the first 4-days postoperatively when an early oral feeding protocol is chosen. An important argument in support of early oral feeding is related to the continuous production of saliva. Humans produce 1000 mL saliva or more per day, and with its acidic pH and the presence of amylase, it is likely to be more harmful to sutures than water or food. In support of this, Le Flem et al.^24^ showed that starting water on the second day after surgery significantly reduces the rate of PCF.^25^

There are several factors to consider against the use of a nasogastric tube. It may lead to additional patient discomfort and could be a contributing factor towards causing PCF by pressing on the pharyngeal sutures and promoting gastro-esophageal reflux.^17^ Despite these concerns, this survey indicated that nasogastric tubes are the most frequently used devices for nutritional support after TL – 98.6% of the respondents.

A recently published meta-analyses showed that early oral feeding after TL within the first 5-days does not increase the incidence of PCF.^25^, ^26^, ^27^ Conversely, the results excluded patients after salvage operations or following extensive surgeries that required free or pedicled flap reconstruction. Milinis et al.,^26^ in their meta-analysis of 14 studies, including four randomized clinical trials and 10 observational studies, observed that the PCF rate in early compared with late feeding groups was 15.2% vs. 11.7% in the randomized clinical trials (RR = 1.35, 95% CI [0.68–2.7], *p* = 0.40) and 14.1% vs. 20.5% in cohort studies (RR = 1.0, 95% CI [0.76–1.3], *p* = 0.98). Singh et al.^28^ conducted a meta-analysis of 12 studies and observed an overall higher risk of PCF in early vs. late feeding groups (RR = 1.51, 95% CI [1.17–1.96]).

In 1989, Boyce and Meyers^29^ found that 84.5% of surgeons initiated feeding 7 days after surgery. Furthermore, this waiting period was prolonged to 3-weeks or longer by 65% of specialists in cases of previous radiotherapy. In Brazil, following TL, 78.3% of the respondents still choose to wait at least 7-days to introduce water, while 87% also wait at least until the 7^th^ postoperative day before starting a liquid, mixed, or free diet. We found that 94.6% of participants delay the resumption of feeds in patients who underwent previous radiation or flap reconstruction. The results of a comparative analysis showed that there were significant differences in the reintroduction of feeds after more extensive surgeries and when radio(chemo)therapy or flaps were required. In support of this, most participants cited previous radio(chemo)therapy as the most important factor to delay oral feeds; a complex reconstruction of the pharynx was the second most important factor. The literature (Fagan, 2019) in favor of early resumption of feeds does not include complicated scenarios of patient management in its analyses.^30^ Ideally, further studies should be performed to precisely define patient cohorts where early oral feeding protocols may be instituted.

The answers obtained from the survey reveal that Brazilian head and neck surgeons perform TL mainly in patients without prior radio or chemotherapy. This differs from the data of the international survey, which indicated a larger percentage of groups performing salvage surgery. Organ preservation protocols are more common in North America, Europe, and Oceania compared to surgery, which is the first-line treatment in Africa, the Middle East, Asia, and South America. However, because of the weight of the Brazilian response in the international survey, South American practice is strongly influenced by the conduct of Brazilian head and neck surgeons. The number of surgeries performed each year did not lead to any statistical difference in the feeding decision, once participating groups performing TL in larger numbers have the same approach of those with smaller number of surgeries. This can be explained by the limited evidence available in favor of early feeds in cases requiring laryngopharyngectomy with flap reconstruction or in more advanced cases. We also believe that surgeons who perform a small number of laryngectomies tend to follow the feeding protocols of referral centers, where surgeons perform a larger number of TL.

We did not find any difference regarding the rate of PCF declared by surgeons and the timing of oral feeds after TL. Our findings on complication rates should be considered with caution since these reported rates were not verified. These differences may be explained by the lower percentage of salvage laryngectomies conducted in Brazil compared with those in other regions. The international study found that surgeons who performed more salvage operations reported higher complication rates, specifically regarding PCF.

Finally, our study shows that surgeons who discharge their patients within the first 14 postoperative days allow feeds before the 10th day more frequently than others. These results seem to agree with studies suggesting that early oral feeding is associated with reduced length of hospital stay, despite the lack of any proven causal link. Indeed, there is still need to determine whether the resumption of feeds is really a limiting factor in discharging a patient after TL.

## Conclusion

Most surgeons who responded to this survey choose to postpone the beginning of oral feeds for a minimum of 7-days after TL. The initiation of feeds occurs later in patients who require salvage surgery after radiotherapy and after total laryngopharyngectomy with reconstruction when compared with TL alone. The advantages of early feeds and the selection of patients who may benefit from them require further studies. Notwithstanding, a growing number of publications on the subject show no clear proof to support or refute the decision to follow an early feeding protocol following TL. This group of Brazilian head and neck surgeons follows a cautious strategy when restarting feeds after TL, as observed in other Western countries.

## Financial support

None.

## Declaration of competing interest

The authors declare no conflicts of interest.
